# A Versatile Method for Depth Data Error Estimation in RGB-D Sensors

**DOI:** 10.3390/s18093122

**Published:** 2018-09-16

**Authors:** Elizabeth V. Cabrera, Luis E. Ortiz, Bruno M. F. da Silva, Esteban W. G. Clua, Luiz M. G. Gonçalves

**Affiliations:** 1 Natalnet Associate Laboratories, Federal University of Rio Grande do Norte, Campus Universitário, Natal RN 59.078-970, Brazil; vcabrera@dca.ufrn.br (E.V.C.); lortiz@dca.ufrn.br (L.E.O.); bruno.silva@ect.ufrn.br (B.M.F.d.S.); 2 Institute of Computing, Fluminense Federal University, Campus Praia Vermelha, Niteroi RJ 24.310-346, Brazil; esteban@ic.uff.br

**Keywords:** RGB-D sensors, accuracy, RMS error

## Abstract

We propose a versatile method for estimating the RMS error of depth data provided by generic 3D sensors with the capability of generating RGB and depth (**D**) data of the scene, i.e., the ones based on techniques such as structured light, time of flight and stereo. A common checkerboard is used, the corners are detected and two point clouds are created, one with the real coordinates of the pattern corners and one with the corner coordinates given by the device. After a registration of these two clouds, the RMS error is computed. Then, using curve fittings methods, an equation is obtained that generalizes the RMS error as a function of the distance between the sensor and the checkerboard pattern. The depth errors estimated by our method are compared to those estimated by state-of-the-art approaches, validating its accuracy and utility. This method can be used to rapidly estimate the quality of RGB-D sensors, facilitating robotics applications as SLAM and object recognition.

## 1. Introduction

A typical task that has received great attention in computer vision research is information extraction through the use of sensory data coming from depth sensors. Mainly in the robotics field, these kinds of devices are being used in several applications to capture data from the environment allowing drones or other types of artificial creatures (robots) to perceive the environment and to respond autonomously to visual stimuli coming from the scene [1]. In general, to achieve this behavior, robots embedded computers process such information computing 3D models of the objects in a scene using 2D colored images and depth information of them also called RGB-D data.

Several devices can be used nowadays to determine the depth, such as stereo cameras (Bumblebee, ZED, and Minoru 3D), structured light sensors (Kinect v1, Xtion Pro, PrimeSense, and RealSense) and devices with Time of Flight (ToF) technology (Kinect v2, LIDAR). Despite being widely used in robotics and computer vision, the aforementioned devices can deliver data with errors due to their physical characteristics and algorithms used in the measurement of disparity and/or depth. In the case of stereo cameras, the faults are attributed to the use of lenses with high distortion, poor camera layout and poor resolution for image capture; while in the structured light and ToF sensors the errors mainly appear because of the small range of the captured rate and resolution [2,3,4].

Despite the limitations inherent to each type of sensor, the devices cited above have been used as the basis for many research projects in robotics, mainly because of the relatively low cost and acceptable accuracy. Considering that each sensor has inherent errors, it is important that somehow these are determined or estimated for being treated in the further computations. The error of 3D sensors depth data is a relevant problem in robotics, computer graphics, and virtual reality, among other fields. Different methods are proposed in the literature aiming to solve such problem, particularly, but none that can be extended to most of the devices available in the market. This is one of the reasons that fair comparison between devices is not usually found in the literature.

To overcome this issue, in this paper, we propose a versatile method for computing the depth data RMS error that can be applied for a variety of currently existing sensing devices, or that might be developed in the future, without the need to model geometrically its data capture method. Our approach starts capturing rectified and undistorted images of a common checkerboard, then proceed with the construction of a depth map. The 2D processing of RGB data is performed to find the checkerboard corners, for which 3D coordinates are calculated and two point clouds are created. The first is named as the *estimated cloud* because it refers to the current coordinates of the corners given by the device software. The second is called the *ideal cloud* and is obtained measuring by hand the distances that determine the corners’ position on the checkerboard. A registration between the two point clouds is performed and the resulting RMS error is computed. Finally, with the use of curve fitting methods for interpolation of unknown values, an equation is obtained that generalizes the RMS error as a function of the distance between the sensor and the checkerboard pattern.

These errors curves estimated using our proposed method are compared to those resulting from other approaches [2,5], verifying our methodology. Also, two applications (in simple depth maps correction and point clouds registration) are devised and implemented just to illustrate the utility of the depth RMS errors resulting from our approach. Other applications as wearable sensors [6] or probabilistic robotics [7,8] can also benefit from the RMS error estimation provided by our approach. In this last specific application, previously determined error bounding in function of the distance to points in the environment are used in order to estimate the variance in the depth provided by stereo cameras, which is used for mapping and reconstruction. In the first application [6], some measures of fruit geometric characteristics as diameter, volume, weight (as a function of volume), and skin area, between others, are to be measured from stereo images, and this can also rely on a similar probabilistic procedure.

Therefore, the main contribution of our work is the versatile approach for verifying the error, which can be adopted and easily applied to generic devices. The introduction of a simple method for finding the error curve as a function of the distance between the pattern and the devices is a consequence of this contribution. Also, this work constitutes an excellent tool for the testing of disparity and depth generation algorithms in the construction of 3D vision devices. Finally, because of its versatility, the proposed method can be used to rapidly estimate the quality of RGB-D sensors, benefiting applications such as RGB-D-based SLAM.

In the following sections, we present a theoretical background, followed by an analysis of the related works, and by the necessary methodology for implementing our proposal, the experiments using three devices with different technologies (Kinect v1, v2, and ZED). Finally, the results are analyzed and the method validation is presented.

## 2. Theoretical Background

In order to better understand our proposal, some theoretical background on errors (systematic and non-systematic) is introduced in this section. Also, as we devised two applications to validate our proposal, depth data generation and correction, and point cloud registration, an explanation about these two techniques, including algorithms and concepts, will also be explained next.

### 2.1. Systematic and Non-Systematic Errors

According to He et al. [3] the errors that may occur in ToF depth measurements can be of two types: systematic and non-systematic. These kinds of errors classification are well known, as well, in robotics sensing for navigation as introduced by Borenstein and Feng [9] and the same types can be easily extended to other sensors as 3D stereo cameras. In general, systematic errors are caused by the intrinsic properties and the imaging conditions of the camera system [3]. Their form is relatively fixed, and generally, they can be evaluated in advance, and it is possible to have a correction for them implemented in some way. Systematic errors can generally be reduced by understanding them well and providing a good calibration procedure. According to Grunwald [10], dealing with depth measurements without correction is potentially unreliable because a multitude of systematic errors exists such as sensor nonlinearities, signal degeneration, process inhomogeneities, temperature drifts, just to mention a few.

Nonetheless, besides systematic errors that can be identified and corrected in the calibration process, the non-systematic errors exist as well, with statistical errors (such as noise) but also with other kinds of errors that are more difficult to deal with. Non-systematic errors are caused by unpredictable features of the environment [9] and by imperfections of the sensor. In fact, He et al. [3] divide the non-systematic errors into four categories, signal-to-noise ratio (SNR), multiple light reception, light scattering and motion blurring. Actually the first one (SNR) is a measure of error and the last three are sources of errors. In fact, non-systematic errors are a great problem for actual applications, because it is impossible to determine an upper bound for the error when working under general ambient conditions, besides they can be reduced by using redundant sensors. The main aspect of non-systematic error is that it varies, randomly, what makes it difficult to determine a general mathematical model for describing its exact behavior, so it might not be corrected, but an upper bound for it can be estimated mainly when working under restricted ambient conditions. That is, if the environment does not substantially change, estimated values for the error bound obtained with significant statistics can be used with a great probability of success. In this case of dynamical environments, an easy to use procedure for measuring the RMS error as the one proposed in this work would be useful.

Besides a model for the non-systematic error cannot be determined, a limit for it can be eventually estimated and this can be useful in applications as probabilistic robotics, in order to perform visual localization and mapping [7,8]. An application that needs a prior knowledge of the error estimation is the one dealing with mapping and navigation with a stereo camera [7,8]. In this application, the authors need to apply a sensor model in which the variance of the measured elevation $\sigma^{2}$ should be used. In that work, they adopted that σ increases linearly with the distance defined empirically. So the estimation of the RMS error, as will be described in this paper, could be useful for that work, as it can be easily computed.

Also, notice that it eventually happens that not all systematic error is removed or corrected in the calibration. So this residual might come together with the remaining non-systematic errors that cannot be modeled. In the current work, besides we assume that a calibration procedure has been done and the images are rectified, the error is measured as a black-box. What do we measure is the registering error and the major contributor to this error is undoubtedly the error in depth. That is, we are just measuring the input and output error. Notice that this gives a general error that could be the summation of any kind of error. For example, it could be the summation of eventually existing systematic errors, as manufacturing defects in the optical device, bad calibration, bias in the disparity algorithm, with non-systematic errors, as Gaussian errors corresponding to uncertainties in the position of a corner, and numerical errors at the Singular Value Decomposition (SVD)/Iterative Closest Point (ICP), between others.

As said, the major benefit of our proposed method is its versatility, with the possibility of comparing completely different devices as ZED camera with Kinect, or the Minoru 3D camera with a Panasonic 3D HD camera that we have in our lab, or with the Bumblebee (also a stereo camera). This can be done in a rapid and simple way, in order to have an estimated idea about the superior bounding of the error as a function of the distance to the sensor, which can be effectively used, for example, by probabilistic robotics algorithms [7,8], as explained above.

### 2.2. Theoretical Correction of Depth Data Error

In most current applications of computer vision it is necessary to know the distance, for example, between an autonomous vehicle and objects on a highway. An RGB image provides no information about the geometry of the 3D objects present in a scene, and for this reason, the RGB-D sensors were created encoding depth information directly in a 2D matrix known as depth map (2.5-D) or in a list of 3D coordinates in a given reference frame, called point cloud [11]. In order to get depth, the RGB-D sensors can use several techniques such as structured light [5], ToF [12] and stereo vision [13]. None of these technologies is free of faults so they can return or estimate incorrect depth values. Notice that in some cases we have objects with known geometry in the ambient (for example planes or flat surfaces like a road or a side sign). As the depth sensor presents measures with errors, which are not obeying these geometries, then it seems to be possible to perform some correction by using the measured distances in order to get the correct geometries. In fact, this is possible and in the next we use this argument for showing a straight approach for correction of this kind of error, thus demonstrating one applicability of our approach. If a method can be created that is versatile enough to calculate the error e as a function of the depth values ${\hat{Z}}_{j}$ measured by any sensor, then a map D can be corrected by using Equation (1), where S is the error sign matrix for each depth value (v,u) into depth map and can be computed using Equation (2), where $\mu_{D}$ is the mean of the depth values of D.

(1) $$D_{c}=D+e*S$$

(2) $$S=\left[\begin{array}{ccccc} s_{1,1} & s_{1,2} & s_{1,3} & \cdots & s_{1,u}\\ s_{2,1} & s_{2,2} & s_{2,3} & \cdots & s_{2,u}\\ \vdots & \vdots & \vdots & \ddots & \vdots \\ s_{v,1} & x_{v,2} & s_{v,3} & \cdots & s_{v,u} \end{array}\right],\;\mathrm{where}\;s_{v,u}=\left\{\begin{array}{cc} +1, & \mathrm{if}\;D_{v,u}\geq \mu_{D}\\ -1, & \mathrm{otherwise} \end{array}\right.$$

Note that correction using these equations is very simple and is used only to demonstrate the use of the RMS error curves and not to create a robust correction algorithm. In this case, the mean $\mu_{D}$ is sufficient to calculate the error sign S, that is, our correction example is restricted to flat surfaces.

### 2.3. Point Cloud Registration

Registration [14] is the action of aligning two point clouds in a common reference frame. The registration consists in finding the relative pose between a pair of point clouds, that is, the rigid transformation (rotation and translation) that transforms points from one reference frame to the other one. Several applications benefit from point clouds aligned in a common reference frame, as for example SLAM [15] and object recognition [16].

In general, this process can be carried out by a number of classic algorithms, such as direct registration least squares [17,18], Random Sample Consensus (RANSAC) [19], (ICP) [20] or any variant versions of the last two methods [14]. Methods based on least square usually apply (SVD) to solve for the parameters that best fit the data. These methods are more restricted because they assume known correspondences between points: for each point present in a point cloud, its location is known in the other point cloud.

Because this is rarely the case, some algorithm must be used to find feature points and correspondences between features of the two point clouds being registered [21]. The ICP algorithm may be used when point correspondences are unknown. ICP works iteratively by finding correspondences through nearest neighbor algorithms and using these correspondences as inputs to a core rigid transformation method. The algorithm is repeated until the alignment error is below a threshold or the number of iterations reaches a maximum. Alternatively, RANSAC may be employed to simultaneously estimate the registration transformation and classify feature correspondences. The process is also iterative and works by sampling random correspondences, estimating a rigid transformation with the sampled data and verifying how the remaining data fits the candidate transformation. The algorithm is terminated when a prespecified portion of the data agrees with the estimated model or the number of iterations reaches a maximum.

## 3. Related Works

In this section, we show works found in the literature that present methodologies for evaluating and quantifying the depth data error provided by 3D sensors of the three above mentioned technologies: structured light, ToF, and stereo vision. The most representative device based on structured light sensing technique is the first version of the Microsoft Kinect, hereinafter referred to as Kinect v1. Since its inception, this device has been used in various computer vision applications [22,23,24,25] due to its cost and availability. Because of this, there is a number of important studies related to its calibration and accuracy. For example, Khoshelham and Elberink [5] propose a model to determine the accuracy of the sensor considering the estimation of depth data from disparity. A theoretical model of random error of depth measurement is presented and it is determined that there is a quadratic growth of the error as a function of the distance between the camera and the sensed object. To demonstrate this relationship, 3D point clouds are taken from a flat portion of a door and a plane is adjusted to the data, allowing the distance from each point to the plane to be taken as a representation of the random error. Menna et al. [26] present a geometric modeling of the Kinect v1 considering the operational approach of the IR camera-projector and determines a theoretical graph of precision of depth as a function of the distance to an object. Experimentally, the author concludes that the depth values of the edges present high errors. The value of the deviations between 3D points estimated by the sensor and its best-adjusted plane decreases when 20% of the edge pixels are removed.

Since 2014 Microsoft began to distribute the Kinect v2 that has the ToF as its operating principle. After its launch, several authors as Lachat et al. [27] and Yang et al. [12] have worked on the accuracy of this sensor. The first work [27] analyzes photometric and depth data, to determine the depth error considering the average value of a window of 10 × 10 central pixels of a depth image of a wall. For this, a set of fifty maps is captured for each distance between the sensor and the target. The variation between the real distance of the sensor to the wall (measured by tachometry) and the values of depth maps are assumed as the sensor error. In the second work [12], real distances (wall-sensor) are obtained from two laser meters and all pixels of the depth images are taken into account for the error analysis.

The comparison of the accuracy of data provided by 3D sensors has been an aspect commonly addressed in the selection of the capture device to be used in the development of applications such as 3D mapping, visual odometry, and obstacle detection. Several methods to determine the accuracy and precision of sensors that use ToF and structured light technologies can be found at the works of Rauscher et al. [2], Zennaro et al. [28] and Jorge et al. [29]. The first two works [2,28] select data from a depth map that is part of a whiteboard or a flat wall located in front of the sensor. With the 3D data (point cloud) they reproduce the experiment done in [5] and determine the depth error of the sensors. Jorge et al. [29] maintain the idea of using point clouds to determine the accuracy and precision in Kinect V1 and Asus Xtion depth data. The accuracy of these sensors is calculated by comparing the distances between the centers of the spheres (3D coordinates) estimated by the sensors and measurements done with an external device. For captures done at one and two meters the sensors accuracy varies between 5 mm to -15 mm and 5 to -25 mm respectively. Wasenmuller and Stricker [30] consider an error that is the difference between depth values of a flat wall generated by the sensors (depth maps) and their corresponding values in a ground truth of depth. The ground truth is formed by the depths of the corners of a checkerboard, these corners are detected in the IR image and its depth calculated based on the pose of the board determined with the perspective-*n*-point algorithm [31]. The increase in the standard deviation of the differences is exponentially modeled for the two compared devices Kinect v1, v2. Plagliari and Pinto [32] also compare the data measured by the two versions of Kinect, to define the accuracy and precision of the depth measurements given by the devices as a function of the distance to an object. Interpolated functions are presented from data obtained experimentally. The data considered for the analysis is part of a central window of 200 × 200 pixels taken from one hundred depth maps of a wall that is parallel to the sensor. The accuracy of the sensor at each distance corresponds to the average residue between the reference distance (taken from laser meters) and the average of each corresponding pixel, to determine the accuracy of the sensor the average standard deviation is computed for each capture interval.

In relation to devices based on stereo cameras, Jin et al. [33] present an analytic and experimental study guided to determine the error of depth estimation in these sensors. The author assumes that errors are caused by the alteration of the disparity values. So the error in the disparity is attributed exclusively to the lenses distortions of the cameras disregarding errors in the calibration, matching or construction stage of the stereo system. A second-order polynomial mathematical model is proposed to represent the relationship between the estimated depth values and the real values. Oh et al. [34] define a model for the error caused by the stereo matching computation process. The approach considers that the location variation of the same object points in the grid pixel of two stereo images can generate an error from -0.5 to +0.5 pixels in the disparity values, assuming that they are correctly computed. This disparity error causes inaccuracy in depth estimations, considering that the pixel error in disparity values are uniformly distributed from -0.5 to +0.5. The same amount of error is determined in depth data with respect to the focal distance and baseline of the cameras.

Jing et al. [35] determine the accuracy of Kinects and PrimeSense devices. The approach is based on the plane fitting technique already presented in previous works. This paper [35] describes an approach in which a global error for all sensor operation ranges is determined, also considering that the correction of the depth maps can be made with values obtained through linear equations. Smisek et al. [36] calculate the depth data error for Kinect v1, stereo SLR, and the SR-4000 camera. The error is assumed as the variation between the raw data given by the sensor and the reconstructed points in the calibration processes of the cameras. Similar to work [35], it is provided a global error for the devices. These approaches stand aside from our proposal because they do not provide a mathematical model for the error that can be compared with our results.

In a previous work [13] we adopted a more complex approach to verify the error and the maximum distance at which the ZED camera could return reliable values of depth. We tested using several, different patterns, with one checkerboard, and with two or three checkerboards disposed perpendicularly between each other thus acquiring clouds forming a 3D structure. The results of this previous work show that the qualities are not so different using one instead of two or three checkerboards. Also, as reported by their makers [37] the ZED device is fine for distances up to 20 m, besides with that approach we have shown that the error is unacceptable at this distance, with the ZED working fine up to some 15 m. So we decided in this work to use a pattern with just one checkerboard, that is much easier to transport from place to place for the tests, coming up with a more versatile technique. Additionally, notice that one of the other techniques evidenced above to determine the depth error involves the use of a ground truth of depth, which can be obtained using laser meters, a tape or with geometric techniques [12,30,32]. Our proposal is not subject to obtaining any distance measures because all corners of the board have a set value of depth equal to zero. Other methods are based on the plane or sphere fitting [2,28,29], because they are approximations that can be subject to errors attributed to the presence of atypical depth values as well as variations of the fitting. In our proposal, the coordinates of the corners are known and it is guaranteed that there is no presence of errors in their location.

The works mentioned above are concentrated around the Kinect v1 and/or v2 or stereo cameras, individually, but not in all of them at once. Nonetheless, the drawback of these methods is that they have been developed for a specific device and cannot be applied nor have been tested with other sensors. Therefore, the present work relies on our previous work [13] aiming to propose a new and generic solution to the problem, by providing a versatile method that can be used in most 3D devices that are capable of providing an RGB image and its corresponding depth (**D**).

In the literature it is evidenced that the development of algorithms to register point clouds of rigid [38] and non-rigid objects [39] is the focus of several research studies. The works of Khoshelham et al. [40] and Santos et al. [41] propose to improve the accuracy of the results obtained from the register using the error model of the sensor. They specifically show that assigning weights based on the theoretical random error [5] of the depth measurements improves the accuracy of pairwise registration and sensor pose estimates along the trajectory. Also, Nguyen et al. [42] estimate the Kinect v1 depth error model and used it to improve the 3D object reconstruction and pose estimation.

## 4. Versatile Approach for Depth RMS Error Estimation

The goal of our work is to quantify the variation between a depth value estimated by an RGB-D sensor and its ideal value (the ground truth). Because no measurement device is entirely accurate, errors in depth measurements of a sensed object are prone to increase with the distance to the object. Ultimately, this leads to general errors affecting the captured geometry of the scene deteriorating, thus, the performance of computer vision applications, such as visual odometry [43,44] and object recognition [16].

To quantify this variation we propose a versatile method, despite also being simple and easy in practice. We justify the characteristics of the method as follows. Firstly, the method is versatile because it can be employed to assess any RGB-D sensor, independently of its underlying depth sensing technology (e.g., structured light, ToF, etc.). Moreover, the method is simple in its conception because it relies solely on the three-dimensional error estimation, i.e., the error model is computed in 3D space instead of being evaluated in 2D (e.g., disparity) image coordinates, as is the general case of related works (e.g., [5]). Lastly, the method is practical because its requirements are a laser ruler and the use of a planar checkerboard pattern, as commonly adopted by camera calibration methods [45]. The laser ruler is not used for measuring the distance to corners as previous works. Instead, it is used to ensure that the RGB-D device has its image plane parallel to the wall having the checkerboard pattern, while the pattern serves as the ground truth to our method.

**Figure 1:**
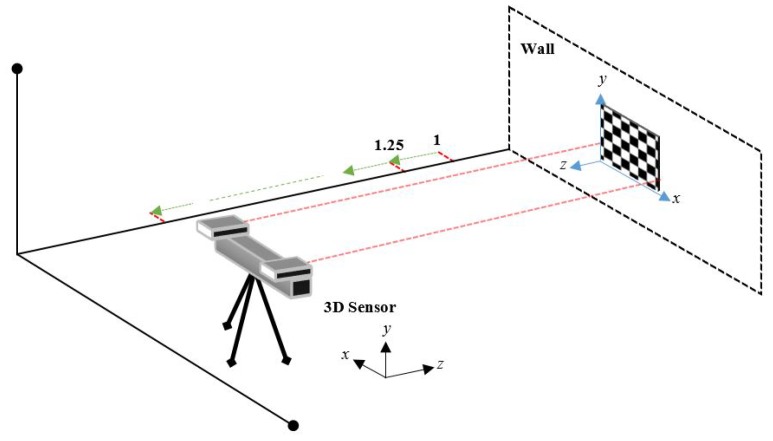
Data acquisition scheme.

Assessing the error of an RGB-D sensor with our method involves placing a checkerboard pattern on a flat wall and positioning the sensor in front of and pointing toward the wall. The parallelism between the device and the wall is ensured by certifying that the camera principal axis is parallel to the normal vector of the wall plane. Figure 1 illustrates this process, which should be repeated for various distances.

The errors computed by capturing images of the checkerboard at several distances from the sensor are the basis for generating a parametric model of polynomial or exponential type representing the RMS depth error throughout its operating range. Notice that our proposal is robust to errors originating from the estimation of planes for the quantification of the depth error (as performed by previous works [2,5]) since the ideal points are inside a checkerboard with a completely rigid and smooth surface.

### 4.1. Assumptions

**Figure 2:**
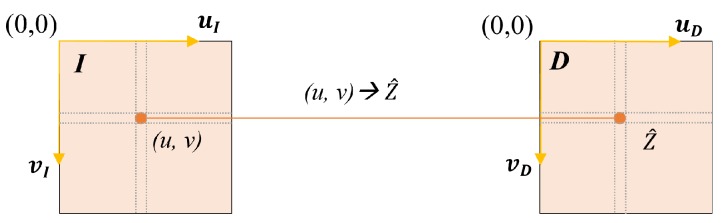
Correspondence between RGB image and depth map.

The proposed method assumes that the RGB and depth images are rectified (without distortions) and registered. More specifically, given an RGB image *I* and associated depth image *D*, the column/row pair (u,v) indexes the appearance I(u,v) and the depth D(u,v) of a single physical 3D point p present in the captured scene that is projected on image coordinates (u,v), as shown in Figure 2.

The 3D point p is represented by its ideal coordinates ${\left[X,Y,Z\right]}^{t}$ in a world fixed reference frame, while $\hat{p}={\left[\hat{X},\hat{Y},\hat{Z}\right]}^{t}$ denotes the estimated 3D coordinates given by the RGB-D sensor of the same point in a camera fixed reference frame. Due to the projective nature of RGB-D cameras, $\hat{X}$ and $\hat{Y}$ are computed as a function of the depth $\hat{Z}$, as given by Equation (3). In this equation, $f_{x}$, $f_{y}$ denote the focal distance in pixels along the horizontal and vertical directions and $C_{x}$ and $C_{y}$ are the coordinates of the projection center. These parameters are referred to as the camera intrinsic parameters and are obtained by calibration procedures [45].

(3) $$\begin{array}{cc} \hat{X}=\frac{u-C_{x}}{f_{x}}\hat{Z}, & \;\hat{Y}=\frac{v-C_{y}}{f_{y}}\hat{Z} \end{array}$$

### 4.2. Point Cloud Generation

In order to estimate the error in depth data for a generic RGB-D sensor, we proceed with an evaluation that computes the alignment error in the 3D space. For this, two tridimensional planar point clouds are constructed: the point cloud $I=\{p_{1},p_{2},..,p_{N}\}$ of ideal points and the point cloud $E=\{{\hat{p}}_{1},{\hat{p}}_{2},..,{\hat{p}}_{N}\}$ of estimated points.

The ideal point cloud I is generated with the aid of the checkerboard pattern and all of its points are referenced in a coordinate system with origin fixed in the plane of the checkerboard. For a checkerboard having a total of N=ST points arranged in a rectangular grid of *S* rows and *T* columns, the point cloud I is generated as $\left\{{\left[0,0,0\right]}^{t},{\left[d,0,0\right]}^{t},{\left[2d,0,0\right]}^{t},...,{\left[0,d,0\right]}^{t},{\left[0,2d,0\right]}^{t},...,{\left[\left(T-1\right)d,\left(S-1\right)d,0\right]}^{t}\right\}$, where *d* is the size of the checkerboard square.

**Figure 3:**
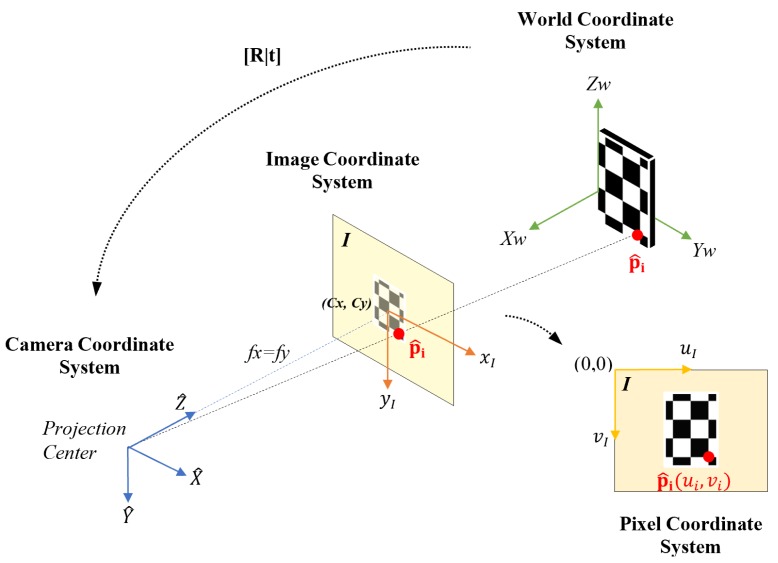
Image projection of the ideal and estimated point.

The estimated point cloud E is generated by detecting corners on the checkerboard intersections. Specifically, the image coordinates $\left(u_{i},v_{i}\right)$, i=1,2,...,N of each corner point are automatically detected by checkerboard corner detection algorithms [46]. Using the 2D coordinates $\left(u_{i},v_{i}\right)$ as indices to access the depth map *D*, the estimated depth ${\hat{Z}}_{i}=D\left(u_{i},v_{i}\right)$ of the point ${\hat{p}}_{i}$ is collected, allowing the other two coordinates ${\hat{X}}_{i}$ and ${\hat{Y}}_{i}$ of ${\hat{p}}_{i}$ to be computed by plugging $\left(u_{i},v_{i}\right)$ and ${\hat{Z}}_{i}$ in Equation (3). This process is shown in Figure 3.

The operating range of certain RGB-D sensors, notably those of structured light and some ToF sensors [47], is relatively short (approximately 0.5 to 5 m). In contrast, stereo cameras may have an operating range of up to 20 m, as is the case of the ZED [37]. Our proposal aims to analyze the RMS error over all the operating ranges of the sensors, and thus, it is essential to detect checkerboard corners in images captured throughout short and long distances.

There are several algorithms to detect checkerboard corners in the literature, as for example those proposed by Bouguet [45] (that are included in OpenCV library) and Geiger et al. [46]. These two approaches detect corners with a sub-pixel precision. However, due to the requirement of our method of long-range detection, we elect the latter approach for this task. While the OpenCV algorithm is able to detect corners in low-resolution images in ranges of 5 m, the algorithm of Geiger et al. detects corners in distances from 0.5 up to 22 m. Furthermore, the mentioned algorithm has other desirable features, such as the successful detection of corners in indoor and outdoor environments and also under varying light conditions.

It could be thought and argued that the corner detection process could contribute to an additional error that is added up to the final depth error of each sensor being evaluated. However, we made experiments not shown here that demonstrate that this is not the case since the RMS reprojection error of the detected corners has values of at most 0.1 pixels with images captured at distances of 20 m from the sensor.

### 4.3. Depth Error Evaluation by Point Cloud Alignment

After generating both the ideal I and estimated E point clouds, the depth error of a given RGB-D sensor can be evaluated. This process is then carried out by computing the alignment error between both point clouds. For this, the two point clouds I and E are firstly registered in a common reference frame. The registration is solved through absolute orientation algorithms [17] that seek the rigid transformation given by the rotation matrix *R* and translation vector *T* minimizing Equation (4).

(4) $$R,T=\underset{\hat{R},\hat{T}}{\mathrm{argmin}}\sum_{i}\left|\right|p_{i}-\left(\hat{R}{\hat{p}}_{i}+\hat{T}\right){\left|\right|}^{2}$$

The algorithm is computed based on the fact that the correspondences between each ideal point $p_{i}$ and its estimated counterpart ${\hat{p}}_{i}$ are known, that is, the index *i* refers to the same point in both point clouds I and E.

After being registered in the same reference frame, the squared 3D error $e_{i}^{2}$ of the point *i* is evaluated by the square of the Euclidean distance between its ideal coordinates $p_{i}$ and its estimated coordinates ${\hat{p}}_{i}$, as shows Equation (5).

(5) $$e_{i}^{2}=\left|\right|p_{i}-{\hat{p}}_{i}{\left|\right|}^{2}={\left(X_{i}-{\hat{X}}_{i}\right)}^{2}+{\left(Y_{i}-{\hat{Y}}_{i}\right)}^{2}+{\left(Z_{i}-{\hat{Z}}_{i}\right)}^{2}$$

Note that if the estimated point ${\hat{p}}_{i}$ has the same coordinates as its ideal point $p_{i}$, the error $e_{i}^{2}$ is 0, although this is rarely the case. More specifically, there are no errors in the matching process between points and therefore, the error $e_{i}^{2}$ is directly related to the 3D coordinates estimated by the sensor ${\hat{p}}_{i}$, which in its turn encompasses the depth error of the point.

The alignment error $E(Z^{j})$ for a given distance $Z^{j}$ between both point clouds is then computed by summing the squared errors $e_{i}^{2}$ for all i=1,...,N points, as shown in Equation (6).

(6) $$E\left(Z^{j}\right)=\sum_{i=1}^{n}e_{i}^{2}$$

### 4.4. RMS Error Model Estimation

An error model for the RGB-D sensor being evaluated is estimated by varying the distance between the sensor and the checkerboard pattern. With this model, it becomes possible to quantify the general behavior of the device error for known and unknown (interpolated and/or extrapolated) distance values.

For this, the RMS error is calculated for various distances $Z^{j}$, j=1,..,M. The RMS error is then calculated as the square root of the mean of the sum of quadratic errors $E(Z^{j})$, as given by Equation (7). In this equation, $E(Z^{j})$ is computed over the ideal $I^{j}$ and estimated $E^{j}$ point clouds generated at distance $Z^{j}$.

(7) $$e_{RMS}^{j}=\sqrt{\frac{1}{n}\;E\left(Z^{j}\right)}$$

With several values for $e_{RMS}^{j}$, it is possible to estimate the parameters of two types of continuous error curves, using an exponential or a polynomial interpolation function. The exponential error model, shown in Equation (8), has two parameters *a* and *b*, while the polynomial error model (Equation (9)) has three parameters *a*, *b* and *c*.

(8) $$f_{1}\left({\tilde{Z}}^{j}\right)=ae^{b{\tilde{Z}}^{j}}$$

(9) $$f_{2}\left({\tilde{Z}}^{j}\right)=a+b{\tilde{Z}}^{j}+c{\tilde{Z}}^{j\;2}$$

Both models are computed for the average distance ${\tilde{Z}}^{j}$ of all the *n* points ${\hat{p}}_{i}$, that is

$${\tilde{Z}}^{j}=\frac{1}{n}\sum_{i=1}^{n}{\hat{Z}}_{i}.$$

Finally, the parameters of both models are calculated by least squares curve fitting after gathering *M* pairs of values $\left({\tilde{Z}}^{j},e_{RMS}^{j}\right)$ for all set distances j=1,...,M.

## 5. Experiments and Results

In order to demonstrate the versatility of our RMS error estimation method, three devices are used: Kinect v1, v2, and ZED. With each of them, the depth data is captured by varying the distance between the sensor and the test object. For the Kinect (v1, v2) the captures vary from 1 to 4 m and for the ZED up to 7 m, all of them with a capture interval of 0.25 m. To ensure that the sensor and the checkerboard are parallel, two laser distance meters (Bosch GLM80) take place at the extremities of the 3D sensors, as seen in Figure 1.

### 5.1. Finding the RMS Error

The 3D projection of the corners detected in the RGB images involves the use of the intrinsic parameters of the sensor. Because in the Kinect (v1, v2) the IR camera is considered to be the origin of the adopted coordinate system in respect to which the three-dimensional points are represented, it is necessary to use its parameters and that means that there is a correspondence between the IR and RGB images. The programming tools Libfreenect and Libfreenect2 [48] provide the intrinsic factory parameters of Kinect v1, v2, respectively. The parameters of the ZED camera are available from the manufacturer. We use the ones from the left camera because it is considered to be a reference in the depth calculation. These parameters are seen in Table 1.

The checkerboard used as a test object has twenty-eight internal corners (4 × 7) separated by each other by 150 mm (as seen in Figure 3). To reduce the effect of noise in the depth maps generated by the devices we capture 300 frames for each target-sensor distance.

**Figure 4:**
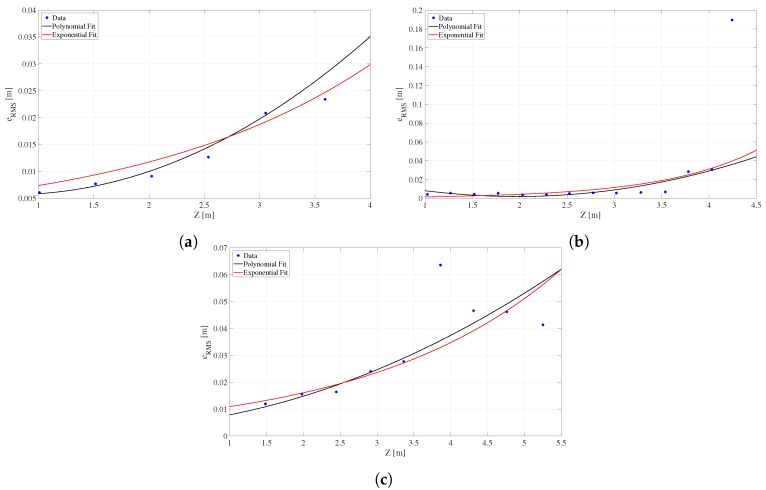
RMS error for (**a**) Kinect v1, (**b**) Kinect v2 and (**c**) ZED, represented for polynomial and exponential models.

With the depth and RMS error values, the mathematical models for the Kinect (v1, v2) and the ZED are obtained. Observing the trend and behavior of the RMS error data in Figure 4, it can be noticed that the above (polynomial or exponential) interpolation models can be used to continuously represent this error. However, only a visual analysis is not enough, so the goodness-of-fit statistics were used to numerically determine which is the best model: the Sum of Squares Due to Error (SSE), $R_{square}$, and fit Standard Error (*S*) [49]. We consider the best-fit criteria that SSE and *S* are close to zero, and $R_{square}$ has a value close to one [50]. The Table 2 shows all of goodness-of-fit statistics for the polynomial and exponential models to fit the RMS error for each one three devices. For the Kinect v2 and the ZED camera, the best model is an exponential type represented by Equation (8). In the case of the Kinect v1, the best adjustment is achieved by means of a second-order polynomial defined by Equation (9), validating what was presented in previous work [2]. The Table 3 lists the calculated coefficients (*a*, *b*, *c* for polynomial and *a*, *b* for exponential model) for each device.

### 5.2. Validation

In this section we start by demonstrating the accuracy and usefulness of our method, providing a comparison of our RMS error curves with other ones that we found in the literature [2,5]. Specifically, a comparison of the trend of the curves is made. In the next, we demonstrate the usefulness of our method and results in two other experiments. In the first one, we obtain (with the Kinect v1, v2, and ZED) depth maps of a specific scene, and we make a correction of all the depth values in each map and then compare them with the original maps to visualize their level of correction. In the second experiment we capture some point clouds (from the corners of a chessboard), correct them using the depth RMS error and perform their registration (with known correspondences) in pairs. A comparison between the results of the registration using clouds with and without correction is then provided.

#### 5.2.1. Comparison with Other Methods

The analysis of the Kinect v1 depth data error has been approached in several works of the literature, nevertheless each author proposes variants in the methodology of error determination and its representation. The models of Rauscher et al. [2] and Khoshelham and Elberink [5] present the error in a similar way to that made in our proposal, so it is useful to realize a comparison that allows validation of the results obtained with our method.

**Figure 5:**
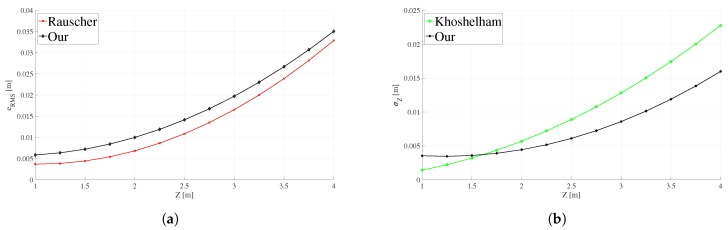
Comparison between methods to estimate the depth error in the Kinect v1. (**a**) Comparison of our method with Rauscher approach [2], using the results of the depth error in terms of RMS ($e_{RMS}$), and (**b**) comparison of our proposal with Khoshelham approach [5] using the standard deviation ($\sigma_{Z}$) to represent the error.

**Figure 6:**
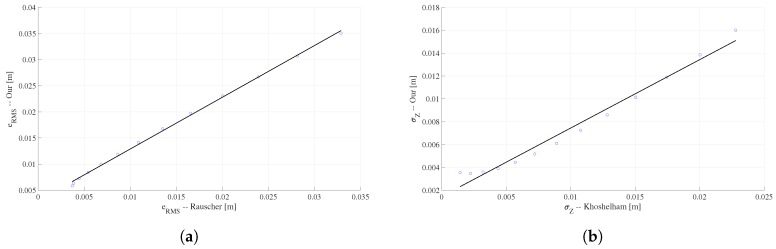
Dispersion graphs of Kinect v1 depth errors curves, (**a**) Rauscher et al. [2] vs. our RMS error ($e_{RMS}$) and (**b**) Khoshelham and Elberink [5] vs. our standard deviation of the depth error ($\sigma_{Z}$).

In the work of Rauscher et al. [2] the error curve has a tendency very similar to that obtained in our proposal, which is shown in Figure 5a. It is observed that when the sensor-target distance increases the two polynomial curves tend to overlap. The scatter diagram of Figure 6 shows that the error values of the two models vary linearly so that the quantification of their degree of relationship can be calculated by the correlation coefficient. The coefficient obtained is 0.99, this implies that the two curves have a strong positive relationship, therefore the curve obtained with our proposal correctly represents the depth RMS error of the Kinect v1 and the method of this proposal can be satisfactorily used in other 3D sensors.

The Khoshelham and Elberink [5] proposal consider a standard deviation representation of the depth error as a function of the distance to the target. Then, before making the comparison it is necessary to calculate the standard deviation of the error $e_{i}$ (Equation (5)) in each sensor-target distance using our method and adjust a new curve as shown in Figure 5b. The correlation coefficient between the two standard deviation curves of the error is 0.98 which means that the two curves have the same tendency, therefore, also in this case our method allows correct representation of the depth error.

#### 5.2.2. A Simple Depth Map Correction

First, we capture 300 depth maps D of a flat wall located at 1.5 m from the sensors (Kinect v1, v2, and ZED). Then we correct these maps using Equations (1) and (2) with $e=f_{1}\left(D\right)$ (Equation (8)) for the ZED and Kinect v2, and $e=f_{2}\left(D\right)$ (Equation (9)) for the Kinect v1. Note that correction using these equations is very simple and is used only to demonstrate the use of the RMS error curves and not to create a robust correction algorithm, the mean $\mu_{D}$ is sufficient to calculate the error sign S, that is, our correction example is restricted to flat surfaces. Finally, an average of the depth RMS error for each pixel on the 300 depth maps before and after correction is computed. For visualization purpose and because the used depth sensors have different resolution the comparison of depth errors are presented for a central window of 150 × 150 pixels.

**Figure 7:**
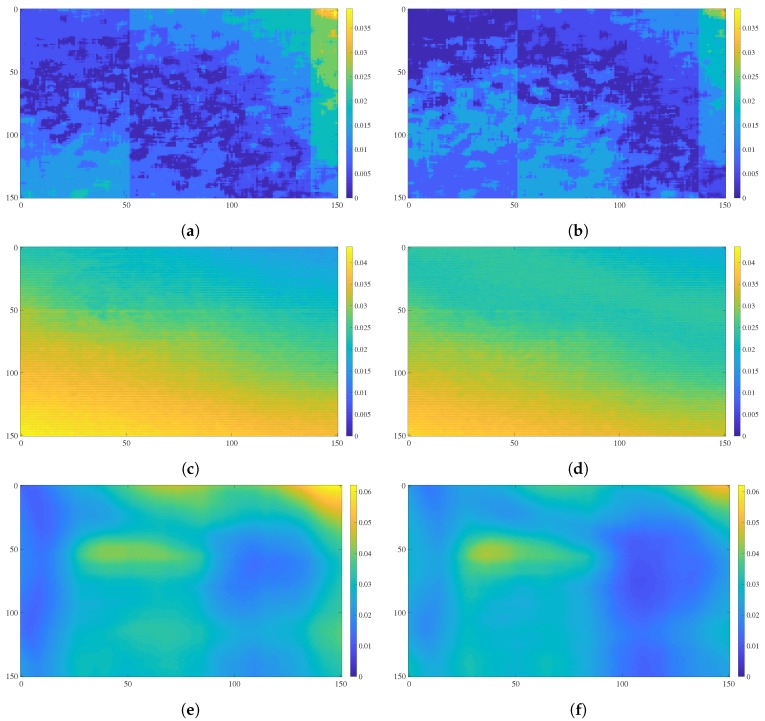
Comparison of the average RMS error in meters (for each pixel in a central window of 150 × 150) of 300 depth maps captured with Kinect v1 (640 × 480 px), Kinect v2 (512 × 424 px) and ZED camera (672 × 376 px), (**a**,**c**,**e**) before and (**b**,**d**,**f**) after a simple correction process. The more yellow the pixels are, the more RMS error exists.

In Figure 7 it is observed that after the correction step there is a decrease in the RMS error, in the case of the Kinect v1, v2 the maximum error decreased by 7 and 2 mm respectively, while in the ZED it is 10 mm. The RMS error color representations of the Kinect v2 shown in Figure 7c,f are similar due to the average correction that is 1.5 mm. This behavior is attributed to the fact that at the selected capture distance (1.5 m) the sensor estimates the depth with a very high accuracy and the RMS error is approximately 3 mm.

#### 5.2.3. Using RMS Error in Point Cloud Registration

**Figure 8:**
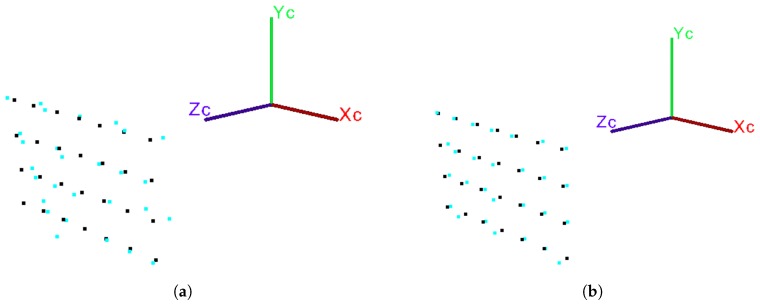
Registration example of two point clouds with known correspondences (captured with the Kinect v1), (**a**) before and (**b**) after correction. The source point cloud (black) is captured at 0.5 m and the target cloud (cyan) is taken at 4 m away from the sensor.

Another useful application of the depth RMS error is in the point cloud registration. To illustrate this, we carried out an experiment that consists of capturing several point clouds (with known correspondences) with the same procedure, as illustrated in Figure 1. The capture range between each pair of clouds is 0.5 m. Performing the registration is done by finding a rigid transformation using the algorithm presented by Arun et al. [17]. The first cloud captured (0.5 m) is considered the source and the following are considered the target. The correction of point clouds is similar to that reported in the Section 5.2.2. In Table 4 it can be seen that after the correction of the point clouds, the RMS error in its registration decreases between 10% and 15%. These results show that a registration between two corrected clouds has less error than a registration with uncorrected clouds. In Figure 8 an example of the effects that the application of the depth RMS error has on the point clouds and on their registration is shown.

### 5.3. Comments on the Experiments and Discussion

The depth values estimated by the Kinect (v1, v2) can be affected by factors related to the environment and the captured object. The natural light as well as the presence of strong flashes alter the values of disparity. For this reason, the chosen scene is illuminated uniformly with fluorescent lamps. Also, it is noticed that the shiny surfaces of certain objects affect the device, which leads to incorrect disparities [5]. For this reason, we use a checkerboard with no bright areas and whose material is not light-absorbing [12]. In order to ensure that the Kinect (v1, v2) depth data is reliable it is necessary to respect the sensors warm-up time of 30 min. Also, even after that, the depth images captured in the first three seconds are not included in our analysis due to the noise that they may present [27].

By comparing the 3D sensors (Kinect v1, v2, and ZED) in light of out methodology, it can be noticed that for applications in which it is intended to be used data of depth of up to 3.5 m it is preferable to use Kinect v2 because its RMS error is smaller than ZED and Kinect v1. For greater distances, the ZED should be used because it obtains data with the lowest depth RMS error.

## 6. Conclusions

The versatile method proposed in this work shows that the determination of the RMS error in depth values estimated by 3D sensors can be made for generic devices from data obtained from a planar checkerboard. Through the implementation of this method, it was possible to calculate and to compare the RMS error for three of the most used sensors in computer vision and robotics applications, the Kinect (v1, v2) and the ZED camera. Notice that this is done without the need for complex mathematical modeling, which involves knowing the specific operating characteristics or technologies for each device.

The equal or better results obtained when comparing the depth errors estimated with our method against those exposed in other works found in the literature allowed validation of the accuracy of the method. Besides the versatile method for error estimation, a practical example of correction of depth maps was also developed in this work based on the mathematical models of estimated RMS error for the 3D sensors, with a simple pixel to pixel correction applied to each map. These are the main contributions of the work, besides making these non-previously reported data available to the computer vision and robotics community for the three devices at a glance.

Future works are planned to demonstrate the utility of the obtained results and the applicability in more complex tasks involved in robot mapping and navigation using SLAM algorithms. These include methods for visual SLAM based on solving the quadratic eigenvalue problem that will be developed using these results. However, in the very near future, an extension of this method that will be carried out is to determine the precision of the used sensors in robotics applications in which the robot is in motion. To do that, as the robot passes through the checkerboards (or landmarks with known positions/orientation), the robot positioning is determined with respect to those and the estimated coordinates compared with the real (ideal) coordinates of these landmarks.

## Figures and Tables

**Table 1:** Intrinsic parameters for the tested sensors.

	Kinect v1 (640 × 480 px)	Kinect v2 (512 × 424 px)	ZED (672 × 376 px)
***fx***	522.259	366.435	338.054
***fy***	523.419	366.435	338.054
***Cx***	330.18	259.478	356.882
***Cy***	254.437	203.774	174.081

**Table 2:** Goodness-of-fit statistics analysis.

Device	Fit Model	SSE	$R_{\mathrm{square}}$	*S*
Kinect v1	**Polynomial**	1.522 × $10^{-6}$	0.99	0.00071
	Exponential	0.0001848	0.97	0.00608
Kinect v2	Polynomial	0.0001175	0.88	0.00361
	**Exponential**	5.778 × $10^{-5}$	0.94	0.00268
ZED	Polynomial	3.996 × $10^{-5}$	0.97	0.00282
	**Exponential**	3.529 × $10^{-6}$	0.99	0.00108

**Table 3:** Coefficients for best fit models.

Device	Fit Model	Coefficients
Kinect v1	Polynomial	a = 0.002797; b = -0.004249; c = 0.007311
Kinect v2	Exponential	a = 0.0005877; b = 0.9925
ZED	Exponential	a = 0.007437; b = 0.3855

**Table 4:** RMS Error in millimeters for the rigid registration, before and after correction of the point clouds.

Distance Cloud-to-Cloud (mm)	Kinect v1		Kinect v2		ZED	
-	Before	After	Before	After	Before	After
500	5.27	4.48	5.57	4.74	8.89	7.55
1000	9.21	7.83	6.53	5.55	13.89	11.81
1500	11.91	10.13	7.76	6.60	14.79	12.57
2000	18.76	15.95	8.85	7.52	21.85	18.57
2500	23.04	19.58	11.68	9.93	23.17	19.69
3000	24.24	20.61	12.67	10.77	48.23	41.00
3500	48.85	41.52	15.98	13.58	61.28	52.09

## Abbreviations

The following abbreviations are used in this manuscript:

ToF	Time of Flight
RMS	Root Mean Squared
SSE	Sum of Squares Due to Error
*S*	Standard Error
